# Cranialization of the Frontal Sinus for Secondary Mucocele Prevention following Open Surgery for Benign Frontal Lesions

**DOI:** 10.1371/journal.pone.0083820

**Published:** 2013-12-23

**Authors:** Gilad Horowitz, Moran Amit, Oded Ben-Ari, Ziv Gil, Abraham Abergel, Nevo Margalit, Oren Cavel, Oshri Wasserzug, Dan M. Fliss

**Affiliations:** 1 Department of Otolaryngology, Head & Neck and Maxillofacial Surgery, Tel Aviv Sourasky Medical Center, Tel Aviv, Israel; 2 Department of Otolaryngology and Head & Neck Surgery, Rambam Medical Center, Haifa, Israel; 3 Department of Neurosurgery, Tel Aviv Sourasky Medical Center, Tel Aviv, Israel; Boston University, United States of America

## Abstract

**Objective:**

To compare frontal sinus cranialization to obliteration for future prevention of secondary mucocele formation following open surgery for benign lesions of the frontal sinus.

**Study Design:**

Retrospective case series.

**Setting:**

Tertiary academic medical center.

**Patients:**

Sixty-nine patients operated for benign frontal sinus pathology between 1994 and 2011.

**Interventions:**

Open excision of benign frontal sinus pathology followed by either frontal obliteration (n = 41, 59%) or frontal cranialization (n = 28, 41%).

**Main Outcome Measures:**

The prevalence of post-surgical complications and secondary mucocele formation were compiled.

**Results:**

Pathologies included osteoma (n = 34, 49%), mucocele (n = 27, 39%), fibrous dysplasia (n = 6, 9%), and encephalocele (n = 2, 3%). Complications included skin infections (n = 6), postoperative cutaneous fistula (n = 1), telecanthus (n = 4), diplopia (n = 3), nasal deformity (n = 2) and epiphora (n = 1). None of the patients suffered from postoperative CSF leak, meningitis or pneumocephalus. Six patients, all of whom had previously undergone frontal sinus obliteration, required revision surgery due to secondary mucocele formation. Statistical analysis using non-inferiority test reveal that cranialization of the frontal sinus is non-inferior to obliteration for preventing secondary mucocele formation (*P*<0.0001).

**Conclusion:**

Cranialization of the frontal sinus appears to be a good option for prevention of secondary mucocele development after open excision of benign frontal sinus lesions.

## Introduction

Cranialization of the frontal sinus, as first described in 1978 by Donald and Bernstein, [1] consists of eliminating the posterior wall of the frontal sinus, meticulous removal of the frontal sinus mucosa, and allowing the frontal lobe come to rest against the anterior table and floor of the frontal sinus. [2] Consequently, the area originally occupied by the frontal sinus is left as dead space or filled with free adipose tissue. [1], [3], [4] Unlike cranialization, obliteration of the frontal sinus does not involve the removal of the posterior wall of the frontal sinus, but rather the meticulous removal of all visible mucosa and the inner cortex of the sinus wall, permanent occlusion of the frontal recess, and the physical obliteration of the sinus. [5], [6] Numerous materials have been advocated for the obliteration of the frontal sinus, including fat graft, rotational flaps such as the pericranial flap, muscle, bone and inorganic materials (e.g., hydroxyapatite cement, bioactive glass and Proplast). [6]–[16] Following open surgery for excision of benign frontal sinus lesions, cranialization would appear to be redundant to conclude the procedure since the posterior wall of the frontal sinus is usually left intact. On the other hand, a relatively high percentage of secondary mucocele formation (ranging from 6% to 25%) following obliteration of the frontal sinus is still reported. [17], [18]

The aim of this study is to compare frontal sinus cranialization to obliteration and to determine if cranialization is inferior to obliteration in terms of secondary mucocele formation and post-surgical complications.

## Methods

Between 1994 and 2011, a total of 69 consecutive patients had undergone excision of benign frontal sinus pathology by open procedures followed by one of two surgical approaches, obliteration (type A osteotomy) of the frontal sinus (n = 41, 59%), and cranialization (type B osteotomy) of the frontal sinus (n = 28, 41%).

All the study patients were evaluated preoperatively by a multidisciplinary team comprised of a head and neck surgeon, a neurosurgeon and a head and neck radiologist. The radiology evaluation included computerized tomography (CT) and magnetic resonance imaging (MRI). Surgery was conducted with the patients under general anesthesia and while lying in a supine position. The patient's hair was not shaved. [19] Obliteration of the frontal sinus was initially carried out with the use of abdominal fat (n = 18), which was later replaced by hydroxyapatite (n = 6) and eventually by a pericranial flap (n = 17). This was done after meticulous removal of all visible mucosa and the inner cortex of the sinus wall. The cranialization technique consisted of drilling out the posterior wall of the sinus, repositioning the earlier osteotomized segment in its original anatomical place and fixing it with pre-bent titanium plates. All patients received broad-spectrum antibiotics peri-operatively, most commonly a combination of cefuroxime sodium and metronidazole hydrochloride. All surgical wounds were drained with two Jackson-Pratt® No. 7 drains, with the penetration wound being concealed behind the ear lobe. Continuous lumbar drains (LD) to prevent transient hydrocephalus and CSF leak, and for monitoring intracranial pressure was not routinely used since they reportedly expose the patients to drainage-related bacterial meningitis. [20]

Following surgery, the patients were admitted to the neurosurgical intensive care unit and treated with steroids and anticonvulsive medications. Any LD′s that had been placed were removed between 48 to 72 hours after surgery. Routine blood tests and follow-up examinations were performed, and the results were documented in the patient's medical files for monitoring intra-operative blood loss and postoperative electrolyte disturbances. In cases of fever, severe headaches, photophobia or changing consciousness, a lumbar puncture was performed and broad-spectrum antibiotics were empirically started and adjusted in accordance with bacterial culture results. Patients presenting with new neurological deficits or deteriorating consciousness underwent emergent CT scanning to rule out impending tension pneumocephalus. Routine cultures were obtained, and empiric treatment was started in cases of suspected wound infection. Suspected ocular complications were evaluated, followed-up and treated by in-house ophthalmologists. Steroid treatment was tapered and antiepileptic medications that had been administered for the first time during the index surgery were also tapered throughout the 2–3 postoperative weeks. Drains were extracted when daily output decreased to <25 cc and the surgical clips were removed on the tenth postoperative day. After discharge from the hospital, the patients were followed-up in our Base of Skull Outpatient Clinic. MRI is routinely performed three months following surgery and annually thereafter to assess recurrent cases and complications, such as secondary mucocele formation.

The Tel-Aviv "Sourasky" medical center institutional review board (IRB) has approved retrospective analysis of all personal files without the need to obtain an informed consent with the obligation that private information shall not be disclosed and that participants shall remain incognito including limitations on recognizable facial features in intra-operative photos.

### Statistical Analysis

Statistical comparison between obliteration and cranialization of the frontal sinus was performed using non-inferiority testing with a pre-defined absolute margin difference of 0.2. Statistical analysis was performed using SAS software version 9.2 (SAS Institute Inc, Cary, NC).

## Results

The records of all consecutive cases of benign lesions of the frontal sinus were extracted and the retrieved data were analyzed. Data on demographics (**Table 1**), type of resected pathology (**Table 2**), postoperative complications (**Table 3**) and length of follow-up, as well as cases of revision surgery (**Table 4**) were available for all the reported 69 cases. Forty-three males and 26 females were operated: 23 males and 18 females were in the obliteration group, and 20 males and eight females were in the cranialization group (*P* = .29). The average age was 35 years for the obliterative group and 34 years for the cranialization group (*P* = .75).

**Table 1:** Patient Demographics.

Demographics	Obliteration (n = 41, 59%)	Cranialization (n = 28, 41%)	*P* value
Mean age, y	35	34	NS
Gender	23M/18F	20M/8F	NS
Mean follow-up, mo. (range)	66 (41–138)*	49 (35–95)*	0.06

Not all subjects are currently in active follow-up.

**Table 2:** Pathologies.

Lesion	Obliteration (n = 41, 59%)	Cranialization (n = 28, 41%)	*P* value
Osteoma	22	12	NS
Mucocele	15	13	NS
Fibrous dysplasia	4	2	NS
Encephaocele	0	1	NS

**Table 3:** Complications.

Complications	Obliteration (n = 41, 59%)	Cranialization (n = 28, 41%)	*P* value
Skin infection	4	2	NS
Telecanthus	3	1	NS
Diplopia	2	1	NS
Nasal deformity	2	0	NS
Epiphora	1	0	NS
Cutaneous fistula	0	1	NS
Total	12 (29.2%)	5 (17.8%)	= .82

**Table 4:** Revisions.

Case	Age, y	Gender	Pathology	Initial approach	Years to failure	Revision approach
1	28	M	Osteoma	Obliteration	1	Obliteration
2	31	M	Mucocele	Obliteration	2	Cranialization
3	33	M	Mucocele	Obliteration	5	Cranialization
4	19	M	FD	Obliteration	5.5	Cranialization
5	29	M	Osteoma	Obliteration	6	Cranialization
6	26	M	FD	Obliteration	9	Cranialization

Abbreviation: y, years; FD, fibrous dysplasia.

The pathologies included osteoma (n = 34, 49%), mucocele (n = 27, 39%), fibrous dysplasia (n = 6, 9%) and encephalocele (n = 2, 3%). Statistical analysis revealed no significant differences between the pathologies of the patients in the two groups (*P* = .14). Surgical complications included skin infections (n = 6, 8.7%), postoperative cutaneous fistula (n = 1, 1.4% [on day 14]), telecanthus (n = 4, 5.7%), diplopia (n = 3, 4.3%), nasal deformity (n = 2, 2.9%), and epiphora (n = 1, 1.4%). There were no cases of postoperative CSF leak, meningitis or pneumocephalus. The overall rate of complications was 24.6%: it was 29.2% (12/41) in the obliteration group and 17.8% (5/28) in the cranialization group (*P* = .82) (**Table 3**).

Six patients required a surgical open revision after a secondary mucocele had developed (prior to the routine use of endoscopes in our department or in cases that where inaccessible by endoscopic surgery): all six patients had initially undergone obliteration of the frontal sinus (14.6% of the obliteration group). The median time for revision surgery was 4.3 years (**Table 4**). The revision was a sequential obliteration in one case, and the obliteration was modified to a cranialization of the frontal sinus in five cases. None of the patients has required a second revision to date. The mean follow-up time was 66 months (41–138 months) for the obliteration group and 49 months (35–95 months) for the cranialization group (*P* = 0.06).

Statistical analysis revealed that cranialization is non-inferior to obliteration in terms of preventing secondary mucocele development (*P* non-inferiority<0.0001, *P* equivalence = 0.0355).

### Comment

During the past two decades, the enhancement of the endoscopic endonasal approach (EEA) has led to a conceptual change, with most benign frontal sinus pathologies being approached trans-nasally. Nevertheless, there are recognized constraints that preclude treatment of all patients with benign frontal sinus pathologies via an EEA (e.g. anterior, lateral, intracranial and orbital extensions), [21] and those patients will undergo open procedures. Our group has gained considerable experience in obliterating the frontal sinus following open surgical excisions. [22] Retrospective analyses of cases involving surgical obliteration of benign lesions disclosed good results, with <15% (n = 6) failed cases requiring revision surgery as a consequence of secondary mucocele development. Although these results are in agreement with similar large series that reported a 6% to 25% failure rate after obliteration of the frontal sinus, [17], [18] we looked for other surgical options that might yield better outcomes, among them restoration attempts of frontal sinus functionality, as described by Lothrop in 1914. [23] Unexpectedly, almost all of our attempts at functional restoration had failed due to postoperative fibrosis and obstruction of the reconstructed frontal recess, including futile attempts to use various stents for long periods of time, as previously reported by us. [24]

Accumulating experience in the use of frontal sinus cranialization after excisions of malignant pathologies and trauma cases [25], [26] convinced us that it is a safe and efficacious surgical technique. Initial results had led us to consider frontal sinus cranialization as the default solution after excision of benign lesions of the frontal sinus when open approaches were indicated and frontal recess stenosis was expected. Data on 28 cases of frontal sinus cranialization with a mean follow-up time of 31 months revealed that none of the patients who underwent cranialization of the frontal sinus had either a secondary mucocele or a postoperative intracranial complication, including five failed cases of frontal sinus obliteration that were modified to cranialization. These results can probably be explained, at least in part, by the pathophysiological mechanisms that are activated following frontal sinus obliteration. Obliteration of the frontal sinus includes filling the sinus with various materials, as previously described, after the meticulous removal of all visible mucosa and the inner cortex of the sinus wall and iatrogenic obstruction of the frontal recess. However, over time, the materials used to fill the sinus can be absorbed, as had been noted in cases of fat obliteration. [27], [28] Moreover, secondary frontal mucocele may develop since residual respiratory epithelium that had not been completely drilled out secretes mucous in what has now become a closed box. Unlike frontal obliteration, cranialization completely eliminates the frontal sinus, thus obviating this chain of events.

Most publications on frontal sinus cranialization describe trauma cases. Constantinidis et al. [29] reported eight patients who had undergone frontal sinus cranialization, four due to trauma and the other four due to benign lesions of the frontal sinus. None of these patients had serious complications nor did they develop secondary mucocele. Complication rates after frontal sinus cranialization are generally low, with only sporadic cases of postoperative meningitis or pneumocephalus having been reported and no reported cases of secondary mucocele developing over time. [30]–[35] Similar results were described in a recently published paper that covered a mean follow-up of 6.5 years after frontal sinus cranialization that was indicated in cases of long-standing frontal sinusitis failing other remedies (n = 15). [36]

We are aware of the limitations inherent in to retrospective analyses such as ours. Comparisons between trauma- and chronic infection-caused pathologies to benign lesions of the frontal sinus are problematic. The relatively short follow-up time in the cranialization group is a major limitation, given that secondary mucocele is a long-term complication. Another caveat is the fact that this is an historical series: most indications for open surgery that had been valid in the past are now obsolete in light of surgical advances in endoscopic surgery. Indeed, not only do most patients undergo endoscopic surgery for primary benign lesions of the frontal sinus, the vast majority of secondary mucoceles are initially treated with great success via EEA.

## Conclusion

Cranialization of the frontal sinus appears to be a good option for prevention of secondary mucocele development after open excision of benign frontal sinus lesions.
